# MaSC: mappability-sensitive cross-correlation for estimating mean fragment length of single-end short-read sequencing data

**DOI:** 10.1093/bioinformatics/btt001

**Published:** 2013-01-07

**Authors:** Parameswaran Ramachandran, Gareth A. Palidwor, Christopher J. Porter, Theodore J. Perkins

**Affiliations:** ^1^Regenerative Medicine Program, Ottawa Hospital Research Institute, K1H 8L6, Ottawa, Canada and ^2^Department of Biochemistry, Microbiology and Immunology, Faculty of Medicine, University of Ottawa, K1H 8M5, Ottawa, Canada

## Abstract

**Motivation:** Reliable estimation of the mean fragment length for next-generation short-read sequencing data is an important step in next-generation sequencing analysis pipelines, most notably because of its impact on the accuracy of the enriched regions identified by peak-calling algorithms. Although many peak-calling algorithms include a fragment-length estimation subroutine, the problem has not been adequately solved, as demonstrated by the variability of the estimates returned by different algorithms.

**Results:** In this article, we investigate the use of strand cross-correlation to estimate mean fragment length of single-end data and show that traditional estimation approaches have mixed reliability. We observe that the mappability of different parts of the genome can introduce an artificial bias into cross-correlation computations, resulting in incorrect fragment-length estimates. We propose a new approach, called mappability-sensitive cross-correlation (MaSC), which removes this bias and allows for accurate and reliable fragment-length estimation. We analyze the computational complexity of this approach, and evaluate its performance on a test suite of NGS datasets, demonstrating its superiority to traditional cross-correlation analysis.

**Availability:** An open-source Perl implementation of our approach is available at http://www.perkinslab.ca/Software.html.

**Contact:** tperkins@ohri.ca

**Supplementary information:** Supplementary data are available at *Bioinformatics* online.

## 1 INTRODUCTION

Next-generation sequencing (NGS) technologies have revolutionized molecular biology with their unprecedented capacity for genome-wide measurement of protein–DNA interactions, chromatin state changes and transcription levels (Mardis, 2011). Although NGS technologies differ in their details, most of the common platforms work by sequencing large numbers of short-DNA fragments. These fragments may originate, for example, from simple extraction of DNA from a sample of cells, selective extraction based on a chromatin-immunoprecipitation pulldown or reverse transcription of RNA into DNA. When dealing with DNA from an organism that lacks a canonical genome assembly, the sequences can be assembled to create a *de novo* estimate of the genome or transcriptome (Narzisi and Mishra, 2011). When the organism does have a canonical genome, the DNA fragment sequences are typically mapped back to the canonical genome, so that their distribution, and especially sites of enrichment, may be studied (Pepke *et al.*, 2009).

NGS technologies usually do not sequence each DNA fragment in its entirety. Indeed, depending on the size of the fragments, this is typically impossible and is not the intended use of the technology. The best practical alternative offered by typical current technologies is sequencing the fragments starting from both ends. However, most experiments do not take advantage of this option for cost reasons and, instead, choose to sequence only one end of each fragment. Thus, despite having a canonical genome assembly to which one end of each fragment can be mapped, most NGS experiments lack information on the other, unsequenced end of each fragment.

A fundamental step in many NGS analysis pipelines is to estimate mean fragment length, so that we can have at least some idea of the genomic locations of the unsequenced ends. First, this helps in the visualization of the NGS dataset in a genome browser. Each read can be ‘extended’ to the average fragment length and shown as an interval in the browser, giving a more accurate impression of the regions of the genome represented by the DNA sample. Secondly, fragment-length estimation is important for peak-calling algorithms—methods for automatically detecting the genomic regions that are enriched in the sample of DNA fragments (Pepke *et al.*, 2009). In most such algorithms, either the reads are extended to an average fragment length (e.g. Tuteja *et al.*, 2009), or the positive- and negative-strand reads are shifted towards each other by half the estimated fragment length (e.g. Zhang *et al.*, 2008). Indeed, many peak-calling algorithms include a fragment-length estimation subroutine. However, many of these have not been rigorously validated, and different algorithms often produce different fragment-length estimates. Partly, as a result, the enriched regions identified by different peak-calling algorithms can have poor overlap (Wilbanks and Facciotti, 2010) (also see Supplementary Section S1). Yet other peak-calling algorithms require the fragment length as input (e.g. Rozowsky *et al.*, 2009). For all of these reasons, reliable fragment-length estimation is an important problem that has not yet been adequately solved.

Mean fragment length can be estimated in the wetlab, and this is often a part of DNA sample preparation protocols. Fragment length is commonly controlled by the aggressiveness of DNA fragmentation (e.g. duration of DNA sonication) and/or by gel-based size selection. However, such procedures vary by laboratory and even by experimenter, and they often go unreported for public NGS datasets. Moreover, such procedures are typically not highly quantitative, and only result in rough estimated ranges for fragment length (e.g. 200–300 bp), which are not satisfactory.

In this article, we investigate the use of cross-correlation of positive- and negative-strand reads for estimating mean fragment length. By applying this method to a small number of available paired-end datasets, for which mean fragment length can be computed exactly, we show that cross-correlation usually produces an accurate fragment-length estimate. Occasionally, however, estimates are clearly wrong, returning a value near the read length rather than the fragment length. Our key insight is that the mappability of different parts of the genome can introduce an artificial bias into the cross-correlation function, which sometimes results in incorrect fragment-length estimates. Based on this insight, we propose a new approach, called *mappability-sensitive cross-correlation* (MaSC), which removes this bias, allowing for much more accurate fragment-length estimation. We analyze the computational complexity of this algorithm and evaluate its performance on a test suite of NGS datasets, demonstrating its superiority to traditional cross-correlation analysis.

## 2 RESULTS

### 2.1 Disagreements between existing fragment-length estimators

A number of algorithms rely on some form of cross-correlation between positive- and negative-strand read sets to estimate fragment length (details provided later in the text). The intuition behind the use of cross-correlation is straightforward. Imagine, for example, a DNA sample that is the result of a chromatin-immunoprecipitation experiment, in which DNA bound to a particular transcription factor (TF) is pulled down. Let us choose a binding site in the genome and consider the subset of all DNA fragments bound to that site. Assuming each fragment is read from only one end, the positive-strand reads will necessarily occur upstream (to the left) of the binding site, whereas the negative-strand reads will occur downstream (to the right) of the binding site. If the fragments are *L* base pairs long on average, then, assuming symmetry, the positive reads will occur approximately *L*/2 base pairs upstream of the site, and the negative reads will occur approximately *L*/2 base pairs downstream. If we correlate the positive- and negative-strand read densities, they should correlate best when the negative-strand reads are shifted upstream by *L* base pairs, which is equivalent to the mean fragment length.

Different algorithms implement this intuition in different ways. For instance, MACS builds a model by measuring the alignment between the positive- and negative-strand reads in a small subset of high-quality peaks (Zhang *et al.*, 2008). The main drawback of this method is that it sometimes fails because of the unavailability of a sufficient number of paired peaks to build a reliable model. Consequently, MACS sometimes ends up assuming a default fragment length of 200 bp. QuEST executes a similar procedure to MACS where the fragment length is estimated from a set of high-quality enriched regions (Valouev *et al.*, 2008). The strand cross-correlation approach (Kharchenko *et al.*, 2008; Landt *et al.*, 2012) computes the Pearson correlation coefficient between the genome-wide positive- and negative-strand read-density profiles. SISSRs, on the other hand, computes a measure of the average proximity of matching positive- and negative-strand reads (Jothi *et al.*, 2008). Other algorithms, such as Peakseq and Peakranger, do not have a built-in fragment-length estimation subroutine (Feng *et al.*, 2011; Rozowsky *et al.*, 2009). Instead, they require the user to input this parameter.

To formalize the correlation approach, let us imagine a genome of B base pairs in total. For simplicity, we assume that the genome has a single chromosome; a generalization to include multiple chromosomes would be straightforward. For 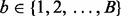, let *f*(*b*) and *g*(*b*) denote the number of positive- and negative-strand reads mapped to position *b* in the genome. We assume these positions represent the ends of the fragments that were read. That is, if a fragment has *R* bases read from one end, and if the *R*-mer that was read maps to positive-strand positions *b* through *b* + *R* − 1, then *f*(*b*) is credited with one read. However, if the same read maps to negative-strand positions *b* through *b* + *R* − 1, then *g*(*b* + *R* − 1) is credited with one read—base position *b* + *R* − 1 represents the physical origin of the start of that negative-strand read. Alternatively, for simplicity, if one prefers to credit *g*(*b*) with a read instead, then one will later have to add *R* − 1 to the fragment-length estimate.

Following what is largely a common practice, we assume that multiple reads mapped to the same position are collapsed into a single read. (Multiple identical reads can be an artifact of PCR amplification; therefore, they are replaced with a single representative, Pepke *et al.*, 2009.) As such, 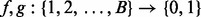. Let us further define 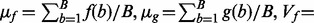

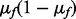
, and 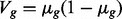. These are just the sample means and variances of *f* and *g*. The cross-correlation of *f* and *g* at a shift (or distance) of *d* is
(1)
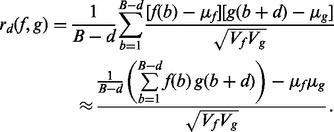



The approximation in the second line results from the summation in the first line not being taken over exactly the same values of *b* as the summations in the formulae for 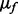 and 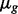. However, in typical cases, 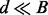, and any difference arising from this approximation would be inconsequential. For a given dataset, we then estimate mean fragment length as 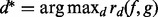, where the 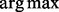 is taken over some plausible range of *d*.

To test whether this whole-genome cross-correlation technique works, we applied it on several *paired-end* datasets downloaded from the GEO database (Barrett *et al.*, 2011). Paired-end datasets contain pairs of reads sequenced from both ends of each DNA fragment. Assuming each end can be mapped back to the genome, the exact length of each fragment and, consequently, the exact mean fragment length, can be computed. For our analysis, a single-end dataset was created from each paired-end dataset (details in Section 4) to hide the paired ends from the algorithm, and the single-end datasets were then used for fragment-length estimation. The results were then corroborated using the true mean fragment lengths. To ensure variety, we chose different types of datasets from different organisms: ChIP-Seq in *Arabidopsis* based on *aborted microspores* (AMS) pulldown, ChIP-Seq in yeast based on a nucleosome pulldown and four RNA-seq datasets from C2C12 mouse myoblasts where the samples have been prepared to have a mean fragment length of either 100 or 280. Because transcript splicing may result in RNA-seq read pairs spanning different exons thereby exhibiting unusually large apparent fragment lengths, we retained only those RNA-seq read pairs with both ends mapping to the same exon according to the UCSC gene definitions. Other processing details can be found in Section 4.

In addition to computing the naïve cross-correlation, we also processed these datasets using four other fragment-length estimation techniques (or subroutines), namely, MACS, SISSRs, the cross-correlation technique by Kharchenko *et al.* (2008) and a technique known as ‘coverage’ that computes the optimal shift for which the number of bases covered by any read is minimized. Although the last technique is briefly mentioned in the ‘chipseq’ R package (Sarkar *et al.*, 2012), we could not obtain a published reference for it. MACS (version 1.4.0rc2) was run directly using Python, whereas the other methods were run using their implementations in the ‘chipseq’ R package, with the command ‘*estimate.mean.fraglen*’.

Table 1 lists the results for the six single-end datasets created from the paired ends, along with the true mean fragment lengths. MACS fails in two of the six cases because of the unavailability of a sufficient number of paired peaks. In the cases where it succeeds, its estimates are accurate. SISSRs demonstrates some variability, and in the majority of the cases, its estimates are far away from the true values. Coverage and Kharchenko-correlation yield accurate estimates for the mouse myoblasts but have difficulties for the *Arabidopsis* and yeast cases. Columns naïve-BC and naïve-WG list the results of our implementations of the naïve cross-correlation, the first on individual chromosomes and the other on the whole genome. Except for the *Arabidopsis* case, our naïve-correlation estimates are within one or two base pairs of the corresponding true mean values. Thus, overall, we can see that the whole-genome cross-correlation works well in many cases but fails sometimes. In the next section, we identify the reasons for failure and propose effective solutions.

**Table 1.** Paired-end mean fragment-length estimates using multiple methods, and the corresponding true mean values

Name	GEO accession	MACS	SISSRs	Kharchenko-correlation	Coverage	Naïve-BC	Naïve-WG	True value
*Arabidopsis* AMS	GSM424618	F	82 ± 28	109 ± 74	209 ± 60	91 ± 76	36	245
Yeast nucleosome	GSM730535	F	12 ± 2	156 ± 6	42 ± 4	153 ± 0	153	154
Mouse myoblasts	GSM582290	96	98 ± 3	100 ± 1	100 ± 0	99 ± 0	99	98
Mouse myoblasts	GSM582293	98	79 ± 2	100 ± 1	100 ± 0	98 ± 0	98	99
Mouse myoblasts	GSM582295	277	148 ± 7	278 ± 1	280 ± 1	278 ± 1	278	277
Mouse myoblasts	GSM582297	285	157 ± 6	286 ± 1	285 ± 1	284 ± 0	284	282

‘F’ indicates failure. Read length is 36 bp for all datasets. SISSRs, Kharchenko-correlation and coverage yield an estimate for each chromosome; therefore, we report means ± 2 × standard errors for these cases. For comparison, mean estimates from naïve correlations computed by chromosome are also shown (naïve-BC). Naïve-WG lists whole-genome naïve correlation results. All estimates have been rounded to the nearest integer.

### 2.2 Problems with naïve cross-correlation as an estimator of fragment length

**Fig. 1.**
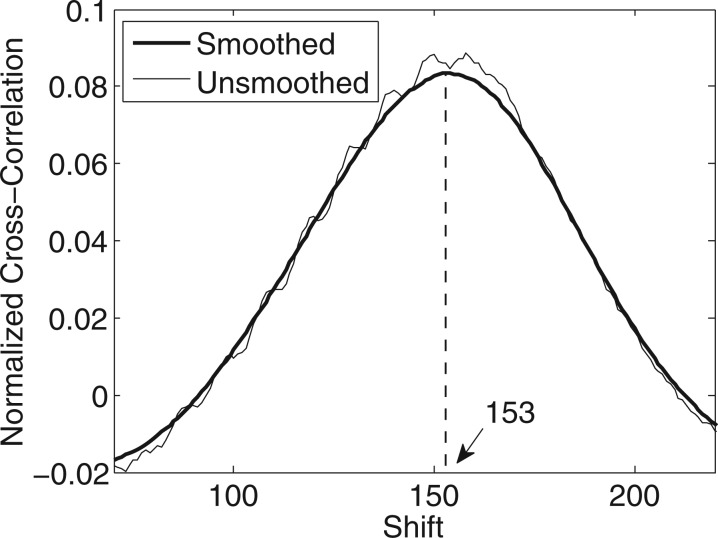
Cross-correlation curve indicating the fragment-length estimate for yeast nucleosome single-end data created from a paired-end dataset. The estimated mean fragment length is 153, whereas the true value is 154. The figure also illustrates the effect of smoothing the correlation curve to more robustly determine fragment length. A central moving average taking 15 samples on either side of the current value has been performed

Two issues are associated with the use of naïve cross-correlation as an estimator of fragment length. The first, relatively minor, issue is the noisy nature of the correlation signal itself because of which there is an uncertainty in the exact location of the peak’s summit. In other words, taking the 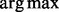 may be overly simplistic. This problem can be circumvented by smoothing the correlation signal using a suitable procedure, such as the moving average filter, thereby obtaining an unambiguous summit location. The effect of smoothing is illustrated in Figure 1, where the raw and the smoothed cross-correlation signals are displayed for the yeast dataset.

**Fig. 2.**
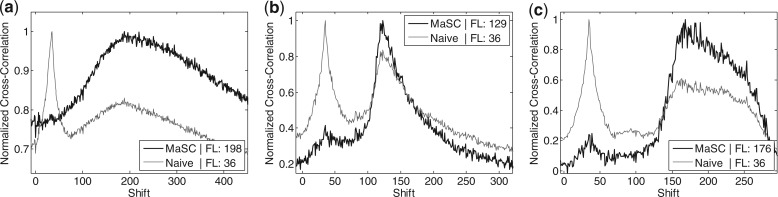
Comparison of fragment-length estimates obtained using naïve cross-correlation and the proposed MaSC algorithm. (**a**) Human HSMMtube ChIP-Seq H3K4me3 pulldown from the ENCODE project (GEO GSM733738), (**b**) mouse lymph node ChIP-Seq EBF1 pulldown (GEO GSM876624) and (**c**) mouse bone marrow input DNA (GEO GSM876641). In all cases, the phantom peak (occurring at the read length of 36 bp) has been eliminated almost completely by the MaSC algorithm, thereby predicting the correct fragment-length estimates. The estimates in the legend insets are the values obtained after applying moving-average smoothing

The other, more serious, problem associated with the method is its unreliability. First, with paired-end data, we observed that the method fails for the *Arabidopsis* dataset. More failures were then encountered on testing further on a number of other single-end datasets, including some from our own group. Still unclear on whether this was just a quirk of the data or a more general phenomenon, we investigated even further using several additional public datasets: ENCODE ChIP-Seq in human based on seven methylation pulldowns, six TF pulldowns and a control (GEO Series GSE29611); ENCODE ChIP-Seq in mouse based on a methylation pulldown, four TF pulldowns and a control (GEO Series GSE31039); and a variety of ChIP-Seq, methylation and input DNA datasets from the EBF1 transcription factor study in mouse (GEO GSE35857) and the histone-modification study in NCCIT cell lines in human (GEO GSE25882). In these datasets too, we found that the naïve cross-correlation sometimes seemed to work and sometimes failed. Figure 2 shows three representative examples where naïve cross-correlation fails (thin curves). The trouble in such cases is caused by the tall ‘phantom’ peak occurring at or around the read length that overshadows the ‘true’ peak around the expected fragment length. A seemingly straightforward solution to this problem is to simply ignore or mask the phantom peak. However, as can be deciphered from Figure 2, the phantom peak not only overshadows the true peak but also affects its shape and location, in many cases displacing the summit significantly. It is, therefore, necessary to systematically address the problem to identify its root cause and thereby eliminate the phantom peak. Although the phenomenon has been partly attributed to poor enrichment (Landt *et al.*, 2012), our investigation revealed other critical mappability issues at play that can be effectively corrected for, resulting in the almost complete elimination of the phantom peak. These details, along with a more complete summary of our analysis, are presented in the next section.

### 2.3 Mappability-sensitive cross-correlation yields correct fragment length estimates on test data

The appearance of the naïve cross-correlation curves in Figure 2 suggests that there are at least two factors influencing the positive- and negative-strand read densities. One results in a peak near the fragment length and the other results in a peak near the read length. We reasoned that the factor related to read length may be occurring because of *mappability*. A given base pair position *b* in the genome is mappable with length *R* reads if the sequence of *R* nucleotides beginning at position *b* occurs nowhere else in the genome. Thus, if a short-read dataset contains a read with exactly those *R* nucleotides, then one presumes that one end of the DNA fragment came from exactly that genomic position. (Although we have assumed an exact match here for simplicity of explanation, mismatches are usually allowed in practice.) However, if the *R*-mer beginning at position *b* matches exactly the *R*-mer beginning at one or more other positions in the genome, then position *b* is deemed to be unmappable. When a dataset contains a read with such an *R*-mer, its true genomic source position is ambiguous; hence, the read is usually discarded. A zero value in the functions *f* and *g* may thus represent either the absence of a read at a mappable position or an unmappable position altogether. If a zero indeed represents an unmappable position, then including it in the cross-correlation computation introduces an artificial correlation between *f* and *g*—in particular, at a shift equal to *R* − 1 because if position *b* is unmappable for a positive-strand read, then position *b* + *R* − 1 is unmappable for the (reverse complement) negative-strand read. We reasoned that if we could correct for this artificial mappability-induced correlation between *f* and *g*, we might eliminate the phantom peak in the cross-correlation curve that confuses the fragment-length estimation algorithm. It must be mentioned that a value of ‘1’ in *f* or *g* has no ambiguity associated with it, as it represents a mappable position where a read has fallen.

**Fig. 3.**
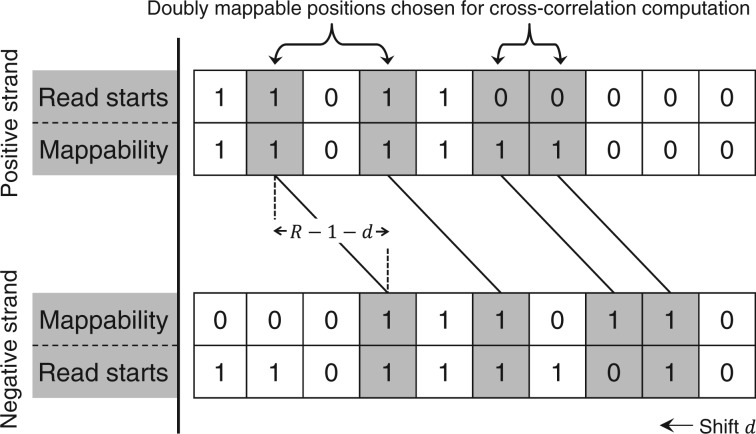
Illustration of MaSC computation at the base-pair level for a single shift *d*. A ‘1’ indicates the presence of a read or a mappable location. Rows 1 and 2 denote positive-strand read locations and mappability, whereas rows 3 and 4 denote negative-strand mappability and read locations, respectively. Only reads falling in doubly mappable locations, i.e. locations having ‘1’s in both rows 2 and 3 (shaded), are included in the cross-correlation computation between rows 1 and 4. Other reads are discarded. Naïve cross-correlation, on the other hand, simply computes correlation between rows 1 and 4, regardless of mappability

The essence of our proposal is to use cross-correlation to estimate fragment length, but to compute it using only bases where the positive strand and the shifted negative strand are both mappable (Fig. 3). More formally, let *M_R_* be the set of positive-strand positions in the genome that are mappable with length *R* reads. This would be the set of shaded positions in row 2 of Figure 3. The corresponding set of positions in the negative strand that are mappable with length *R* reads would be 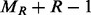, and for a shift *d* of the negative strand, this set would become 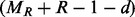. These positions (shaded) are shown in row 3 of Figure 3. Now let 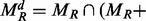 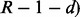, which we call the set of *doubly mappable* positions at shift *d*. If 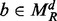, it means that neither *f*(*b*) nor *g*(*b* + *d*) is constrained to be zero because of unmappability. To correct for mappability, the cross-correlation should be computed only over the doubly mappable positions. Accordingly, we define sample means and variances of *f* and *g* as 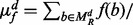 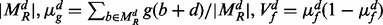 and 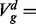 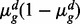, where 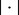 denotes cardinality of the set. The *mappability-sensitive cross-correlation* (MaSC) is then defined as
(2)
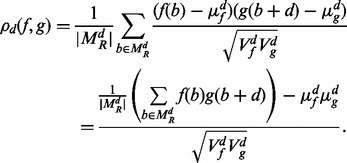

To test whether the MaSC computation corrects fragment-length estimation, we applied it on the single-end datasets mentioned in the previous section. The mappability maps corresponding to the species and read lengths were obtained from the UCSC Table Browser (Karolchik *et al.*, 2004). The thick curves in Figure 2 were obtained using MaSC. From the plots, it is clear that the MaSC algorithm almost completely eliminates the phantom peak occurring at read length, thereby correcting the fragment-length estimate. In ∼40 different datasets we tested, consisting of a variety of ChIP-Seq, methylation and control/input DNA, the phantom peak occurred with varying strengths. In ∼20% of the cases, it was strong enough to overshadow what we take to be the true peak at a plausible fragment-length estimate. In all such cases where the phantom peak presented a problem, MaSC eliminated it almost completely, thus enabling accurate fragment-length estimation. The results for these cases along with the GEO identifiers are presented in Table 2, comparing the fragment-length estimates obtained using naïve cross-correlation, MaSC and the four external methods, namely, MACS, SISSRs, Kharchenko-correlation and ‘coverage’. Since the last three methods output an estimate for each chromosome, for comparison, MaSC results on individual chromosomes are also shown. For these methods, failure rates were computed by setting a threshold that is slightly larger than the read length for a given dataset and classifying all estimates (both chromosomal and means) below this threshold as failures or invalid estimates. These rates are also listed in the table. The threshold was set at 50 for the 36-bp datasets and 90 for the 75-bp dataset.

**Table 2.** Comparison of fragment-length estimates using different methods

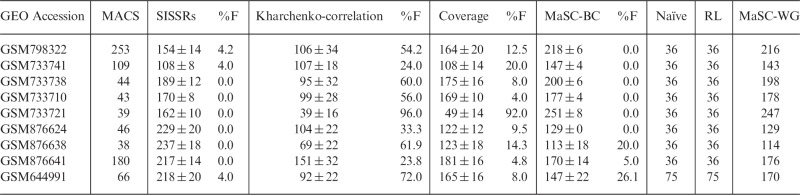

The first five rows correspond to data from the ENCODE project. SISSRs, Kharchenko-correlation and coverage output estimates by chromosome. Hence, these columns have means ± 2 × standard errors. For comparison, MaSC results by chromosome are also included (‘MaSC-BC’). ‘MaSC-WG’ lists whole-genome MaSC results. ‘RL’ lists read lengths. Estimates have been rounded to the nearest integer, and smoothing has been applied for MaSC estimates. The %F columns list the % of failures, i.e. the % of chromosomal estimates at or near the read length.

Since these datasets are single-ended, true mean fragment lengths cannot be computed for benchmarking. Nevertheless, some general observations can be made as follows. MACS yields a valid estimate only in three of the nine cases, whereas in the others it seems to be picking up the phantom peak. SISSRs, on the other hand, yields valid mean estimates with some variability, although, in many cases, its estimates are quite different from those of the other methods. Kharchenko-correlation, which is similar to the naïve correlation, proves to be unreliable for these datasets as expected, displaying large uncertainties and failure rates due largely to the dominance of the phantom peak. The method ‘coverage’, which performed quite well on the paired-end datasets, suffers from significantly large failure rates on the single-end datasets. For the specific case GSM733721, the method completely fails yielding an invalid mean estimate. MaSC-BC performs well on all datasets, including the ones where it has non-zero failure rates. Thus, in the overall analysis, although the concept of strand cross-correlation is well founded and suitable for fragment-length estimation, on its own, it is not sufficiently reliable because of the problems caused by the frequently occurring phantom peak in typical NGS datasets. Tests we conducted using both our implementation as well as that of Kharchenko *et al.* confirm this observation. However, if the causative factor of the phantom peak is corrected for, as we propose here through the MaSC algorithm, the peak is eliminated almost entirely, and the reliability of the cross-correlation technique is greatly improved, resulting in valid and accurate fragment-length estimates. In comparison, none of the other techniques we investigated demonstrated consistent reliability. Between MaSC-BC and MaSC-WG, the latter provides more robust and consistent results. Hence, to obtain accurate estimates, we recommend running MaSC on whole datasets instead of on individual chromosomes. As further tests of robustness, we conducted two more investigations. First, we ran MaSC on a number of randomly sampled subsets of decreasing size of some of our single-end test datasets. MaSC yielded meaningful estimates even with as little as 50% of the reads, although variability in the estimates generally increased with decreasing number of reads (see Supplementary Section S2). Secondly, we used different short-read aligners to remap the reads and ran MaSC on the resulting alignments. The accuracy of the MaSC estimates did not get influenced by the type of the aligner used (see Supplementary Section S3).

### 2.4 Algorithmic implementation and efficiency

In Section 2.3, we have defined MaSC in terms of binary functions *f* and *g*, which indicate the locations of positive- and negative-strand reads. Thus, the most obvious implementation of MaSC would involve computations with such functions. However, NGS datasets are virtually always presented as lists of reads, with each element in a list specifying the position (chromosome and base pair) and orientation (positive or negative strand) of a read. Similarly, information on which positions in a genome are mappable is typically available as lists (Karolchik *et al.*, 2004; Koehler *et al.*, 2011). Thus, a list-based implementation of MaSC would also be natural and possibly more efficient, especially if the lists of reads and mappable intervals are short. To analyze the big-O complexity of these alternatives, let *N* denote the total number of reads, *M* the number of mappable intervals, *G* the genome size and *D* the number of distinct shifts at which we want to compute the cross-correlation.

In a list-based implementation of MaSC, we would begin by separating the list of reads into positive- and negative-strand read lists, *R*^+^ and *R*^−^. We would also ensure that the lists are sorted in ascending order by chromosome and position, so that subsequent operations can be carried out efficiently. Sorting would take 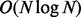 time. Then, for each shift *d*, we would do the following. First, compute the set of doubly mappable base pairs 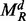 at that shift. This can be done by shifting the mappable intervals to produce 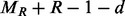, and then intersecting this set with *M_R_*. Both these operations can be carried out in *O*(*M*) time, assuming that the mappable intervals have already been sorted in ascending order. (If the mappable intervals are unsorted, then sorting would cost an additional 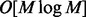 time. However, as this is a one-time cost per organism, we do not include it in our analysis. If the mappable intervals are not available at all, then they need to be computed based on the reference genome. This is a much more significant computation, but again, it is a one-time cost, and, at present, mappable intervals are available for many model organisms at a variety of read lengths.) Secondly, we would intersect the positive- and negative-strand read lists, *R*^+^ and *R*^−^, with the doubly mappable base pairs 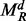, which would take *O*(*N* + *M*) time. At this point, the means and variances in Equation (2) can be readily computed. Finally, to obtain the 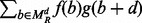 term, we would perform one final intersection, between the positive- and negative-strand reads on doubly mappable bases, which would take *O*(*N*) time. The total time complexity would thus be 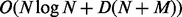.

In a binary-function implementation of MaSC, we would create the functions *f* and *g*, which would take *O*(*G* + *N*) time regardless of whether the original read list is sorted. Then, in *O*(*G* + *M*) time, we would create a function *h* identifying the mappable base pairs according to *M_R_*. Then, for each shift *d*, we would create shifted versions of *g* and *h*, which would take *O*(*G*) time. The various element-wise intersections and summations would also take *O*(*G*) time. The total time complexity would thus be *O*[*D*(*G* + *N* + *M*)].

Thus, in effect, in terms of big-O analysis, the difference between a list-based and binary-function implementations reduces to the difference between an 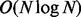 term and an *O*(*DG*) term. As *G* can be in the billions and *D* can be in the tens or hundreds, we would generally expect the list-based implementation to be faster. However, the binary-function implementation can benefit significantly if the functions are implemented as bit vectors, and the shifts and intersections are computed using hardware-level bit-wise operations. Our Perl implementation of MaSC, available at http://www.perkinslab.ca/Software.html, uses bit-vectors. On a single core of a SunFire x2250 computer with 32 Gb RAM, it takes on average ∼30 min to estimate fragment length for each of the datasets studied in this article.

## 3 DISCUSSION

We have demonstrated that mappability can introduce a strong bias into genome-wide cross-correlation computations of positive- and negative-strand read densities. When those computations are carried out to estimate fragment length, and when the bias is strong enough, a dramatically wrong fragment-length estimate can result. When used for peak calling, such incorrect estimates can have adverse effects on the set of peaks returned. Crucially, we have shown that the mappability-induced bias can be corrected for using our MaSC algorithm. Tests using a variety of public NGS datasets demonstrated the effectiveness of MaSC. We also showed that smoothing the correlation signal helps in obtaining an unambiguous summit location. We do recommend checking the plots of the smoothed and the unsmoothed signals provided by our software to ensure a reasonable agreement between the summit locations. The computational complexity of MaSC is comparable with the traditional cross-correlation computation. The only serious caveat to the MaSC computation is that it requires a mappability map to have been established for the target genome and for the read length under consideration. Such maps are not always available and are non-trivial to compute (Koehler *et al.*, 2011). However, when such mappability information is available, we see no reason not to use the MaSC computation instead of the traditional, biased cross-correlation computation.

The pervasiveness of the mappability problem in correlation analysis is unclear. In some datasets we obtained from GEO, we found the problem, whereas other datasets did not show the problem. Generally, we expect datasets with shorter reads to be more susceptible because a greater fraction of the genome is unmappable with shorter reads. However, mappability also varies significantly by organism. The problem seems to be present both in older datasets and new datasets. We are presently planning a comprehensive examination of the large number of ENCODE ChIP-Seq datasets to get a better assessment of the issue.

Although we have emphasized positive- versus negative-strand cross-correlation and the fragment-length estimation problem, our approach to eliminating mappability bias is relevant to other correlative-type analysis of short-read data. For instance, if one were to compute autocorrelation functions as a measure of the spatial structure of the genomic signal being assessed, a similar mappability correction could be performed. The concept could also be relevant to correlating different datasets–for instance, relating a transcription factor binding signal to a histone-modification signal. This would especially be true if the different datasets used different read lengths, and thus were differentially susceptible to mappability problems. Finally, based on the cross-correlation function, we have begun exploring the possibility of estimating not just the mean fragment length, but the variance as well, or, even more generally, the entire fragment-length distribution.

## 4 MATERIALS AND METHODS

A number of preprocessing steps were carried out to prepare the datasets. The paired-end datasets were first used directly to compute the true mean fragment lengths. They were then converted into single-end datasets before applying the fragment-length estimation algorithms. The *Arabidopsis* dataset was available as a single file containing the chromosomal start and end positions of the mapped fragments. However, this file seemed to have been created using paired-end alignment. As paired-end alignments have different mappability profiles compared to single-end alignments, we carried out a fresh alignment (using the same tools as originally used) by randomly choosing one read from each pair and aligning these reads independently as single ends to the TAIR8 genome using SSAHA2. The SAM output from SSAHA2 was converted to a BAM file and then to a BED file using the SAMtools and the BEDtools utilities, respectively, including only hits designated as ‘primary’. The yeast nucleosome data were available as two SRA files, one each for the two ends of the fragments. These were first converted to FASTQ files using the fastq-dump utility from NCBI. The reads were then used in two ways. (i) To obtain the true fragment-length statistics: the reads were mapped to the genome using Bowtie’s paired-end alignment option making sure the FASTQ files met the criteria for a successful paired-end alignment. The resulting SAM file containing the paired-end read locations was converted to BAM and then to a paired-end BED file. (ii) To create a single-end dataset: one read from each pair was randomly selected by walking through the paired FASTQ files, and the chosen set of reads were then independently mapped to the SacCer1 genome (2003) using ELAND (from the CASAVA 1.6 pipeline), including only reads that mapped to a single location (up to two mismatches in the 32-bp seed). The ELAND output was then converted to a BED file using an in-house Perl script. The mouse RNA-Seq datasets were available as BED files containing paired read locations, and since the read pairs had been mapped independently, we could create the single-end dataset by simply subsampling the aligned positions in a random fashion. The only preprocessing steps required were the elimination of orphaned reads by positioning matching reads next to each other and the elimination of read pairs spanning multiple exons.

The single-end datasets were available either directly as BED files, or as SRA or BAM files. In the latter cases, necessary processing, including alignment and format conversions, was performed to obtain BED files. Perl code implementing the MaSC algorithm is freely available at http://www.perkinslab.ca/Software.html.

## Supplementary Material

Supplementary Data
