# Supplementary material for: MaSC: mappability-sensitive cross-correlation for estimating mean fragment length of single-end short-read sequencing data
Source: Bioinformatics. 2013 Jan 7;29(4):444–50. doi: 10.1093/bioinformatics/btt001 (PMC3570216; doi:10.1093/bioinformatics/btt001)
Supplement: Supplementary Data [file supp_29_4_444__index.html]

MaSC: mappability-sensitive cross-correlation for estimating mean fragment length of single-end short-read sequencing data — Supplementary Data 

# MaSC: mappability-sensitive cross-correlation for estimating mean fragment length of single-end short-read sequencing data

## Supplementary Data

files

**Files in this Data Supplement:**

- Supplementary Data - pdf file
