# Supplementary material for: MaSC: mappability-sensitive cross-correlation for estimating mean fragment length of single-end short-read sequencing data
Source: Bioinformatics. 2013 Jan 7;29(4):444–50. doi: 10.1093/bioinformatics/btt001 (PMC3570216; doi:10.1093/bioinformatics/btt001)
Supplement: Supplementary Data [file supp_btt001_Supplementary_File_for_Online_Publication.pdf]

MaSC: Mappability-Sensitive Cross-Correlation for Estimating Mean Fragment Length of Single-End Short Read Sequencing Data

Parameswaran Ramachandran, Gareth A. Palidwor, Christopher J. Porter, and Theodore J. Perkins

Supplementary Material

S1 Effect of fragment length on peak-calling

In this section, we present results that demonstrate the need to have an accurate fragment-length (FL) estimate before calling peaks for NGS datasets. We chose a peak-calling algorithm, namely, MACS version 1.4.0rc2, and a test dataset, namely, FoxA1 ChIP-Seq (available from the MACS webpage), and called peaks for a range of fragment lengths. We also included the FL estimate yielded by our MaSC algorithm. The different peak sets thus obtained were then compared with the peak set corresponding to the MaSC FL estimate of 108. For this comparison, we plotted (shown below) the number of non-overlapping peaks between the peak set corresponding to the MaSC FL estimate and the peak sets corresponding to other fragment lengths.

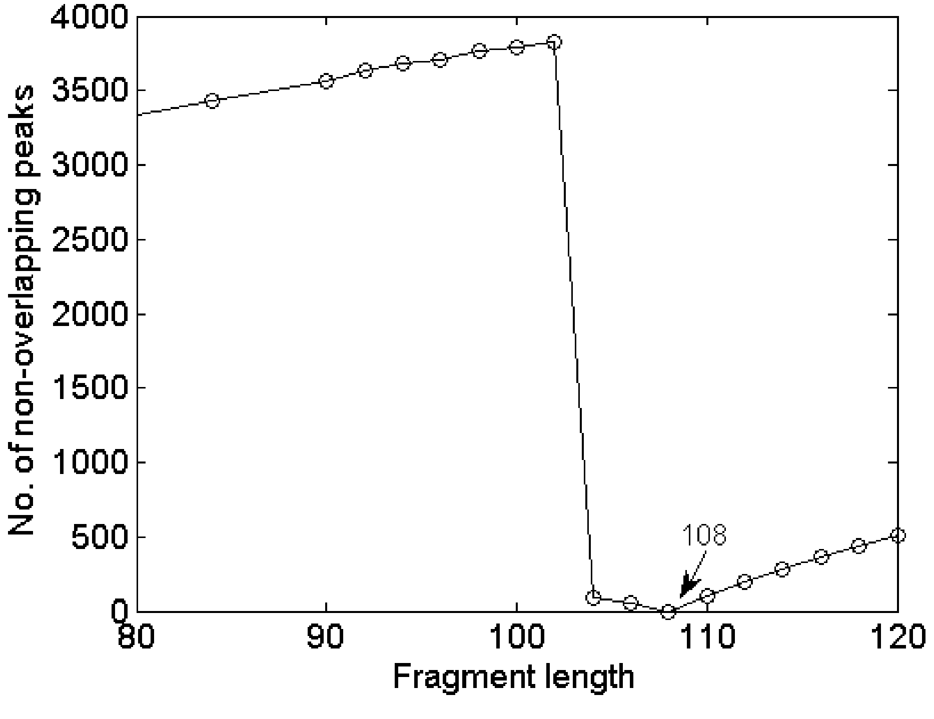

Figure S1: Plot showing the number of non-overlapping peaks between the set of peaks called by MACS using a MaSC FL estimate of 108 and the set of peaks called using a range of other fragment lengths. The dataset used was FoxA1 ChIP-Seq (downloadable from the MACS webpage).

From the above plot, it is clear that the number and the locations of the peaks vary significantly as the fragment-length parameter is varied. Of particular note is the sharp change in the number of non-overlapping peaks when the fragment length is changed from 102 to 104. Although the exact reason for this change requires further investigation, we can conclude that, overall, this analysis emphasizes the importance of obtaining an accurate fragment-length estimate to ascertain a high quality for the peaks called.

S2 Fragment-length estimation using MaSC for fractional datasets

In order to determine the minimum fraction of the total number of reads in a dataset that would be required to obtain a meaningful FL estimate using MaSC, we performed the following analysis for the set of 36-bp single-end datasets listed in Table 2 in the paper. For each dataset, we created subsets containing $x\%$ of reads, where $x \in \{50, 40, 30, 20, 10, 8, 5\}$, by randomly sampling the reads using a uniform distribution from each chromosome. For each value of x, ten different randomly-sampled subsets were created, the MaSC algorithm was run on each subset, and the mean of the resulting FL estimates was taken as the estimate corresponding to that value of x. The results are listed in Table S1. For comparison, the FL estimates obtained using the complete datasets are also shown in the 100% column. This column is reproduced from Table 2 in the main paper.

Table S1: FL estimates for fractional datasets obtained using the MaSC algorithm. Each value shown (except those in the 100% column), is the mean computed across the FL estimates obtained from ten independent randomly-sampled subsets containing the corresponding percentage of the total number of reads.

GEO ID	← Percentage of reads from the original dataset →							
	100%	50%	40%	30%	20%	10%	8%	5%
GSM798322	216	215.4±7.6	221.2±9.3	220.0±13.4	212.0±8.8	207.6±17.3	221.5±16.4	217.8±28.3
GSM733741	143	147.1±3.4	144.7±2.8	146.3±6.0	148.6±8.6	147.3±9.8	145.9±15.8	150.5±17.1
GSM733738	198	197.4±2.8	198.7±6.8	210.8±17.1	206.1±13.2	204.8±15.8	208.2±42.2	213.3±40.9
GSM733710	178	176.0±3.6	176.0±2.9	176.4±6.4	173.9±6.3	175.1±10.7	189.8±11.9	188.2±22.4
GSM733721	247	246.8±11.6	253.3±13.0	247.2±18.6	256.6±16.5	258.9±30.8	248.7±25.7	261.4±45.5
GSM876624	129	128.8±0.4	128.7±0.7	128.8±0.9	128.0±2.2	128.4±3.2	133.0±8.4	127.3±4.6
GSM876638	114	121.5±12.3	138.3±17.6	131.5±16.6	125.4±34.9	112.6±33.4	138.7±44.4	153.3±60.6
GSM876641	176	175.4±3.4	176.5±2.9	176.6±6.9	179.6±8.5	186.0±14.2	191.2±17.1	184.5±12.7

From the table above, we can see that, overall, with decreasing number of reads, the estimates get further away from that obtained using the whole dataset, and they have higher variance. In all cases except GSM876638, a reasonably accurate estimate is obtained even with only 50% of the

reads. For some data sets, considerably fewer reads can be used, although variability in the estimate generally increases as the number of reads decreases.

S3 Comparison of MaSC estimates using different short-read aligners

To test the robustness of the MaSC algorithm against the use of different short-read aligners, we mapped the reads from our 36-bp single-end test datasets using the following tools:

- **Bowtie** using parameters '-l 32 -n 2', which aligns using a 32-bp seed sequence and allows up to 2 mismatches in the seed
- **Bowtie** using parameters '-l 32 -n 2 --best --strata -m 1', which again uses a 32-bp seed and allows 2 mismatches, but also restricts the alignments to one match in the best 'stratum', i.e., with the smallest number of mismatches in the seed sequence. Thus, if there are two genomic locations where the seed has a perfect match, no mapping will be reported (whereas with the other set of parameters, one of the two locations will be chosen at random).
- **Bowtie2** using default parameters (end-to-end mode, which maps the whole read without trimming ends)
- **Burrows-Wheeler Aligner (BWA)** using default parameters

The results are listed in Table S2 below.

Table S2: Comparison of fragment-length estimates obtained after realignments using different short-read aligners.

GEO ID	SRA File ID	Bowtie2	Bowtie (n2)	Bowtie (n2m1bs)	BWA
GSM798322	SRR350915	216	216	216	216
GSM733741	SRR227572	145	143	143	145
GSM733738	SRR227566	198	198	198	198
GSM733710	SRR227504	178	178	178	178
GSM733721	SRR227528	251	251	248	259
GSM876624	SRR408702	129	129	129	129
GSM876638	SRR408716	116	114	114	116
GSM876641	SRR408719	175	175	175	175

As can be seen from the table, five out of the eight datasets tested showed no difference at all in the FL estimates between different aligners, while most of the others showed only minor differences. Thus, we can conclude that using different aligners does not, typically, affect our FL estimates in a significant manner.
